# Polysaccharides with Antitumor Effect in Breast Cancer: A Systematic Review of Non-Clinical Studies

**DOI:** 10.3390/nu13062008

**Published:** 2021-06-10

**Authors:** Claudia Rita Corso, Natalia Mulinari Turin de Oliveira, Leonardo Moura Cordeiro, Karien Sauruk da Silva, Suzany Hellen da Silva Soczek, Virgilio Frota Rossato, Elizabeth Soares Fernandes, Daniele Maria-Ferreira

**Affiliations:** 1 Instituto de Pesquisa Pelé Pequeno Príncipe, Faculdades Pequeno Príncipe, Curitiba 80250-060, PR, Brazil; claudia_rcorso@hotmail.com (C.R.C.); natimulinari@gmail.com (N.M.T.d.O.); leonardocmoura911@gmail.com (L.M.C.); Kariensauruk@outlook.com (K.S.d.S.); suzanyhellen@gmail.com (S.H.d.S.S.); virgiliofrota@gmail.com (V.F.R.); elizabeth.fernandes@pelepequenoprincipe.org.br (E.S.F.); 2 Programa de Pós-graduação em Biotecnologia Aplicada à Saúde da Criança e do Adolescente, Faculdades Pequeno Príncipe, Curitiba 80230-020, PR, Brazil

**Keywords:** polysaccharides, breast cancer, cancer treatment, nonclinical studies, natural source

## Abstract

**Purpose:** To review the effects of polysaccharides and their proposed mechanisms of action in breast cancer experimental models. **Data sources, selection, and extraction:** Articles were selected by using PubMed, ScienceDirect, Scopus, and Medline, assessed from 1 May 2019 to 1 July 2020. The systematic review was registered in the International Prospective Register of Systematic Reviews (Prospero) under the number CRD42020169103. **Results:** Most of the studies explore algae polysaccharides (43.2%), followed by mushrooms (13.5%), plants (13.5%), fruits (10.8%), fungus (2.7%), bacteria, (2.7%), and sea animals (2.7%). A total of 8.1% investigated only in vitro models, 62.1% evaluated only in vivo models, and 29.7% evaluated in vitro and in vivo models. The mechanism of action involves apoptosis, inhibition of cellular proliferation, angiogenesis, and antimetastatic effects through multiple pathways. **Conclusions:** Findings included here support further investigations on the anti-tumor effect of polysaccharides. Some polysaccharides, such as fucoidan and β-glucans, deserve detailed and structured studies aiming at translational research on breast tumors, since they are already used in the clinical practice of other proposals of human health.

## 1. Introduction

The recent advances in research and especially the better access to high-quality care associated with early diagnosis and medical treatment by specialized multidisciplinary teams has improved the survival and quality of life for breast cancer patients [1]. However, breast cancer still represents a significant health problem and is the most common malignancy affecting women around the world, despite being a highly curable disease when detected early [2,3]. Breast cancer incidence differs worldwide and takes into consideration risk factors, availability, and access to mammography for early disease diagnosis. Its incidence is higher in regions with high income (i.e., Australia, New Zealand, North America, and Northern and Western Europe), whereas the lowest incidence is found in low-income regions (i.e., Eastern Asia and middle Africa) [4,5]. In high-income countries, breast cancer is diagnosed at an early stage, often leading to a good prognosis and effective treatment, while in low-income countries, breast cancer is diagnosed later and is consequently associated with higher mortality rates [6].

The exact mechanism by which breast cancer initiates is unknown. However, it is established that the disease is characterized by a heterogeneous molecular expression with different clinical presentations in relation to histology, prognosis, and responsiveness to treatment. The disease is classified in hormonal breast cancer (the estrogenic receptor (ER) and/or progesterone receptor (PR) positive), human epidermal growth factor receptor 2 (HER2) positive, or triple-negative breast cancer (TNBC). Fundamentally, current treatment protocols have evolved to take this heterogeneity into consideration due to their predictive and prognostic implications [7].

Currently, the management of breast cancer is multidisciplinary. Non-metastatic breast cancer treatment goals include eradicating tumors from the breast and regional lymph nodes together with the prevention of metastatic recurrence with radiation therapy. Systematic therapy may be preoperative (neoadjuvant), postoperative (adjuvant), or both, and it includes a variety of treatment strategies based on the clinical pathological features of each case [8].

The systemic therapies include chemotherapy, endocrine therapy for hormone receptor-positive disease, anti-HER2 therapy for HER2-positive cases, as well as the incorporation of bone stabilizing agents, and recently, immunotherapy [9]. Differently, metastatic breast cancer therapy focuses on symptom palliation, improving or maintaining the quality of a patient’s life, as the tumor remains incurable in nearly all affected patients [10].

Despite the great achievements in breast cancer treatment, drug resistance has been observed and continues to be one of the major causes of relapses and death [11]. In this context, many studies have pointed out that natural compounds present a range of biological properties and represent promising options for the discovery of new drugs. In fact, natural products exhibit anti-carcinogenic activity and can target several pathways involved in breast cancer (i.e., cellular proliferation, differentiation, apoptosis, angiogenesis, and metastasis) [12].

Among these products, polysaccharides have attracted attention because of their activity in different types of cancer [13]. Possible mechanisms demonstrated through in vivo and in vitro studies involve cytotoxicity, inhibition of tumor cell growth, apoptosis induction, and immune stimulation [14,15,16,17,18,19,20].

However, despite all the information available in the literature, a systematic review of non-clinical studies referring to the biological activities of polysaccharides in breast cancer is still not present. As information regarding clinical studies is scarce, these studies were not included in this systematic review. Therefore, this review focuses on the effects of polysaccharides from all sources that were studied in the last ten years and their proposed mechanisms of action in breast cancer experimental models. This study aims to provide clear and organized information that can be used as a base for further investigations.

## 2. Methods

This systematic review was carried out following the Preferred Reporting Items for Systematic Reviews and Meta-Analyses (PRISMA) guidelines [21], and its protocol was registered on the International Prospective Register of Systematic Reviews (Prospero) under the number CRD42020169103.

### 2.1. Search Question

PICOS (Participants, Intervention, Comparison, and Outcome) criteria were used to answer the following question: Does polysaccharide treatment have an antitumor effect on non-clinical breast cancer models? (Table 1).

### 2.2. Search Strategy and Study Selection

The search was performed in PubMed, Science Direct, Scopus, and Medline during May 2020 and was updated in June 2020. The search terms considered in this review included “polysaccharides” and “breast cancer” or “breast tumor” or “mammary cancer” or “mammary tumor” and were firstly evaluated in abstracts by two authors (C.R.C. and N.M.T.d.O.). The search strategy was adapted for each database when necessary. Duplicated records were removed by using the Mendeley software. The full-text appraisal was independently assessed by three authors (C.R.C., N.M.T.d.O. and L.C.M.). Discrepancies in any phases were resolved by the senior author (D.M.-F.).

### 2.3. Inclusion and Exclusion Criteria

The search was focused on original non-clinical studies referring to the biological activities of extracts or isolated polysaccharides (e.g., from plants, algae, and fungus) in breast cancer models (e.g., female murine models of breast cancer and breast cancer cells) and was limited to a period of publication (2010–2019) and to articles published in English. Studies in rodent experimental models (rats and mice) of breast cancer with or without metastasis and all breast cancer cell lines were included. Additionally, studies of ex vivo samples (e.g., blood and tumor tissue) of breast tumor models (human or murine) were considered. Only those with doses or concentrations of polysaccharide (isolated or in the form of extracts) below 3000 mg/kg/mL, and with primary (e.g., in vitro: cell viability or proliferation assay; in vivo: tumor weight, tumor volume) and secondary (e.g., biochemical and molecular assays) outcomes were eligible for inclusion.

Articles published in journals with no impact factor, as well as reviews, clinical trials, conferences, book chapters, and studies performed before 2010 were not eligible. Studies with animals with co-morbidities, males, other species rather than rats and mice, and in silico models were not included. Additionally, studies of polysaccharides bound to other compounds (e.g., peptides, antibody, nanoparticles) or studies on adjuvant treatment to chemotherapies were not included in this study.

### 2.4. Data Extraction

Data extraction was performed by four authors (C.R.C., N.M.T.d.O., L.C.M. and K.S.d.S.), and D.M.-F. was consulted to resolve disagreements. The following data were considered:

- Study design: number of experimental groups compared with the vehicle control group.
- Non-clinical models: for in vivo studies: female murine (mice or rats), adult animals (6 weeks of age) with breast cancer, such as xenograft model (e.g., MCF-7, MDA-MB-231 cells) and syngeneic model (e.g., Ehrlich and Walker-256) at any weight body; for in vitro studies: any type of breast cancer cell line.
- Intervention: dose/concentration, time of treatment/incubation, and route of administration when applicable. Type, source, molecular weight, and structure of the polysaccharide.
- Outcome: primary: growth of breast cancer cells; for in vitro: the amount of cell proliferation or viability or migration; for in vivo: tumor volume and/or tumor weight. Secondary: mechanism of action of polysaccharide through biochemical and molecular assay in samples (cells or tumor tissue).

### 2.5. Assessment of Risk of Bias

The methodological quality of each study was assessed considering the risk of bias (RoB) using SYRCLE’s RoB tool for animal studies [22]. The assessment of the risk of bias was performed by five authors (C.R.C., N.M.T.d.O., L.C.M., V.F.R. and S.H.d.S.S.), and D.M.-F. was consulted to resolve disagreements. The assessment included baseline characteristics, allocation concealment, random housing, blinding (performance), random outcome assessment, blinding (detection), incomplete outcome data, selective outcome reporting, sequence generation, and other sources of bias, including the use of statistical analysis, presence of a control group, and complete methodology. Each analysis had three possible judgments (“low risk”, “high risk”, or “unclear risk”). If the information was not clear, the assessment “no information” was selected. For in vitro studies, “no information” was considered as “not applicable” for allocation and random housing. Articles considered to have a high risk of bias in the other source of bias were considered to have overall high risk and were not included in the systematic review.

## 3. Results

### 3.1. Study Selection

**Figure 1:**
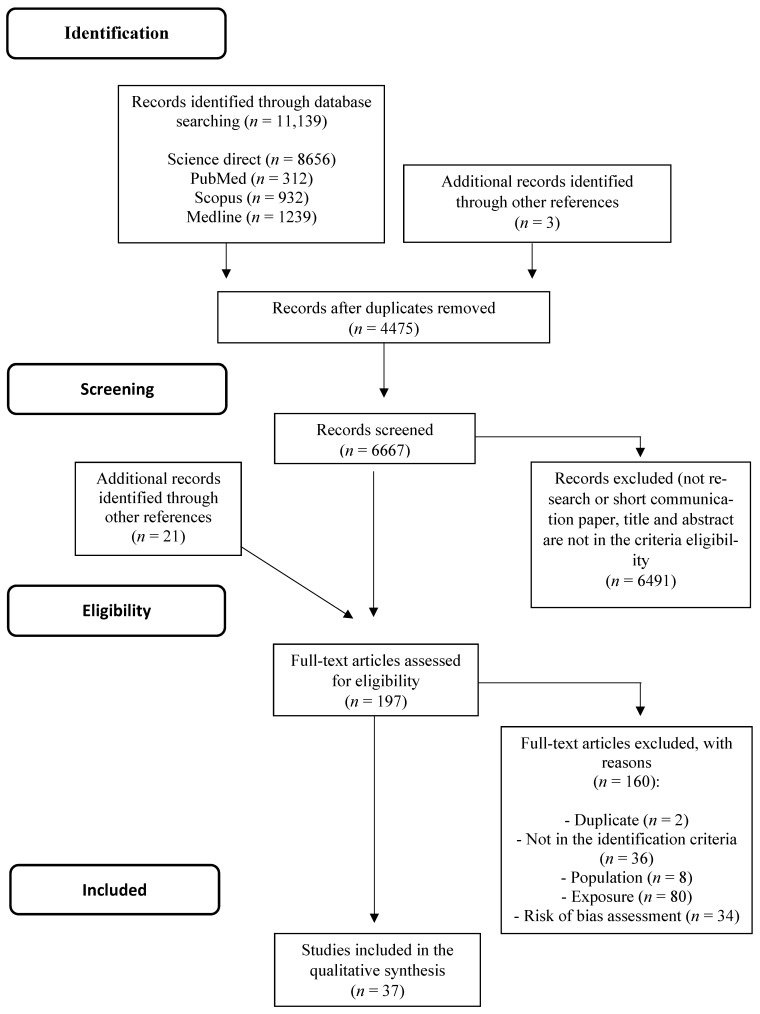
PRISMA Flowchart of the structured literature review.

A total of 11,139 records were identified, including 4475 duplicated records and 6667 records screened by abstract and title. Of those, 6491 were excluded (they were not research or were short communications, or did not fit the criteria following title and abstract analysis). Following scrutiny, a total of 21 articles were included in the identification and screening phases through other references. Then, a total of 197 articles were assessed for eligibility. From this, a total of 160 articles were excluded due to the following reasons: duplicated articles (*n* = 2), articles that did not meet the identification criteria (reviews, articles published before 2010, and articles not in English, *n* = 36), irrelevant population (did not evaluate breast cancer models or used male in vivo models, *n* = 8), irrelevant exposure (did not evaluate polysaccharides per se, *n* = 80), and high risk of bias (*n* = 34). Finally, data analysis was performed in 37 studies. The flowchart of studies selected in the systematic review can be seen in Figure 1.

### 3.2. Risk of Bias Assessment

**Figure 2:**
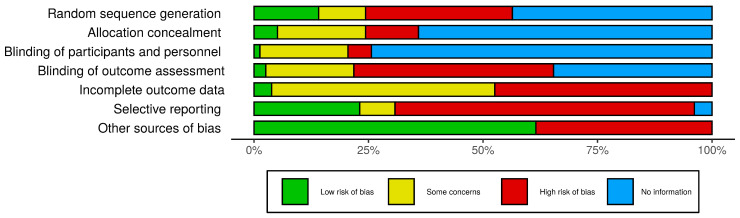
Risk of bias assessment. Evaluation of the methodological quality and assessment of the risk of bias using the SYRCLE’s RoB tool for animal studies. The bars represent the percentage of articles found in each category. Other sources of bias: statistical analysis, presence of control group, or complete methodology. No information also means not applicable for in vitro studies.

Of note, 94.1% of the in vivo studies reported efforts of experimental group randomization, but no one specifically described the randomization procedure. Only one study mentioned that it performed blinding protocol analysis. It is important to point out that 76.3% of the studies were of in vitro protocols. Thus, the random allocation and blinding of participants and blinding outcome assessment domains were considered “not applicable”. Overall, around 47.2% of the studies had no statistical analysis, had no control groups, or presented incomplete methodology (considered as other sources of bias) and were therefore eliminated from this systematic review. The risk of bias assessment is summarized in Figure 2.

### 3.3. Data Extraction

**Figure 3:**
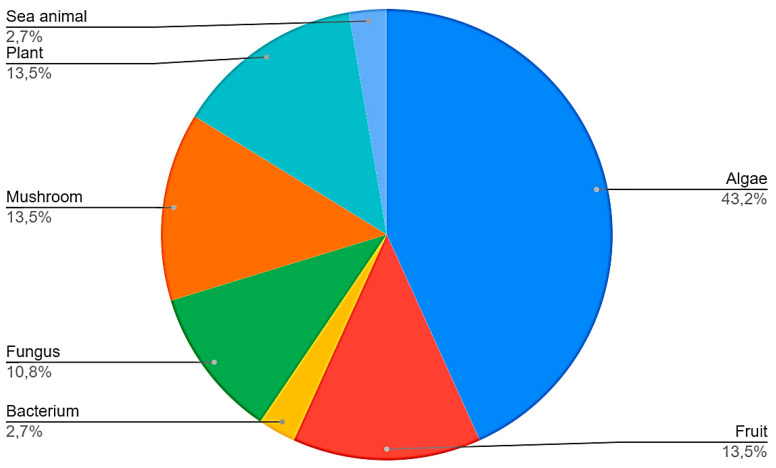
Sources of polysaccharides found in the studies selected in the systematic review.

Sixteen manuscripts (43.2%) analyzed polysaccharides derived from algae, 5 (13.5%) from mushrooms, five (13.5%) from plants, five (13.5%) from fruits, four (10.8%) from fungus, one (2.7%) from bacteria, and one (2.7%) from sea animals (Figure 3). All studies included in the systematic review are summarized in Table 2 [23,24,25,26,27,28,29,30,31,32,33,34,35,36,37,38,39,40,41,42,43,44,45,46,47,48,49,50,51,52,53,54,55,56,57,58,59]. From the 37 articles included in this systematic review, three (8.1%) evaluated only in vivo models, 23 (62.1%) evaluated only in vitro models, and 11 (29.7%) evaluated both in vitro and in vivo models. The anti-tumor effects of each polysaccharide will now be discussed separately by their source of extraction.

### 3.4. Sea Animals

Only one report that extracted polysaccharides from sea animals was found. Lee et al. [23] investigated a starfish polysaccharide (PS) from *Asterina pectinifera*. By employing an in vitro model using MCF-7 cells, they found that PS reduced cyclooxygenase 2 (COX-2) mRNA and aromatase levels in a concentration-dependent manner (10, 20, 40, 80, and 120 µg/mL for 24 h). The expression of MMP-9 was decreased and mRNA expression of tissue inhibitor of matrix metalloproteinase (TIMP)-1, a MMP inhibitor, was increased mainly at 40, 80, and 120 µg/mL. PS reversed phosphorylation of p38, ERK, and JNK [23].

### 3.5. Algaes

From 16 articles, 12 investigated the sulphated polysaccharide fucoidan. Four articles analyzed sulfated polysaccharides from *Laurencia papillosa* and *Undaria pinnatifida*. Three studies with fucoidan were further included in the data analysis after the search term “fucoidan” was used in the databases. Most of the studies with fucoidan (7) were derived from the algae *Fucus vesiculosus*, whereas two were from *Cladosiphon novae-caledoniae* Kylin, one was from *Sargassum hemiphyllum*, one was from *Laminaria japonica* and one study did not mention the source of the polysaccharide. Most of the studies with the fucoidan from *Fucus vesiculosus* (5) investigated in vivo models. Mammary carcinogenesis was induced in rats by using 7,12-dimethylbenz(a)anthracene (DMBA) in two studies, and 4T1 mouse mammary cell linage (a model for stage IV of breast cancer) was used in three studies.

Xue et al. [24], conducted a study to investigate the antitumor effect of the crude fucoidan from *F. vesiculosus*,purchased from Sigma-Aldrich Corporation (St. Louis, MO, USA; MW: 20.000–200.000 kDa) on breast cancer bearing mice and in 4T1 mammary cells. In 4T1 cells, fucoidan inhibited cell proliferation in a time-dependent manner, at the concentration of 50, 100, and 200 µg/mL, for up to 72 h of incubation. Its antitumor effect was associated to a decreased expression of Bcl-2 and Bcl-2 to Bax ratio. The expression of survivin and phosphorylated extracellular signal regulated protein kinases (ERKs), which is required for angiogenesis, was also diminished [24]. A releasing of cytochrome c from the mitochondria into the cytosol and an increased cleaved caspase-3 protein, after fucoidan treatment, were observed. Ten days after tumor inoculation, fucoidan (5 or 10 mg/kg, intraperitoneally, every 2 days, for 10 days) reduced tumor growth and tumor weight. Similarly, a reduction in the number of vessels and vascular endothelial growth factor (VEGF) levels, as well as of apoptosis, was observed in tumor tissue sections [24]. As 4T1 cells have progressively spread metastases to the draining lymph nodes and other organs and are remarkably similar to those of human mammary cancer [60], the authors also investigated the potential of fucoidan in inhibiting metastasis. In fact, both fucoidan treatments (5 and 10 mg/kg) reduced the number of lung metastases. As VEGF can promote tumor cell metastasis, the suppression of VEGF by fucoidan treatment in vivo is likely to be correlated with the antimetastatic effect [24].

To further investigate the mechanisms of fucoidan in protecting against breast cancer, the same group later showed that this molecule is able to inhibit cell growth and increase cell death. Indeed, induction of G1 cell cycle arrest in 4T1 cells was observed. Fucoidan also reduced β-catenin expression and T cell factor/lymphoid-enhancing factor reporter activity and downregulated the expression of apoptosis downstream target genes (c-myc, cyclin D1, and survivin) [25]. Similarly, Hsu et al. [26] found inhibition of cell viability in both 4T1 and MDA-MB-231 cells (60–120 µg/mL for 24 and 48 h). Fucoidan treatment inhibited tumor growth and lung metastasis after 36 days in the 4T1 in vivo model [26]. However, the authors did not give clear information on the dose and route of administration of the compound. Based on the observed antimetastatic effect of fucoidan, the authors investigated epithelial–mesenchymal transition (EMT) markers in 4T1 and MDA-MB-231 cells. Whilst E-cadherin expression was increased, N-cadherin was found to be diminished in these cells. The expression of the transcriptional repressors Snail, Slug, and Twist (EMT, epithelial to mesenchymal transition-related transcriptional repressors), was also reduced.

Indeed, fucoidan (100 µg/mL) presented anti-migratory and anti-invasive effects when incubated with 4T1 and MDA-MB-231 cells, supporting its antimetastatic actions. These were associated with a rapid (1 h) and sustained (24 h) reduction of the transforming growth factor (TGFR) levels, Smad2/3 phosphorylation, and Smad4 expression. The evidence indicates that fucoidan has the potential to inhibit metastasis through regulation of a ubiquitin-dependent degradation pathway that affects the TGFR/Smad/Snail, Slug, Twist, and EMT axes [26].

Chen et al. [27], investigated whether endoplasmic reticulum stress (ER stress) is involved in fucoidan-induced cell apoptosis in the highly invasive and metastatic MDA-MB-231 cells. Fucoidan incubation (50 and 100 ug/mL) reduced the levels of GRP78, a protein responsible for the inhibition of cell apoptosis and the acceleration of ER-associated protein degradation [61]. The same study also showed that fucoidan increases the phosphorylation of calcium/calmodulin-dependent protein kinase 2 (CaMK2) and enhances Bax and caspase 12 levels. Furthermore, fucoidan inhibited the expression of X-box binding protein 1 (XBP1), as a result of decreased inositol-requiring enzyme 1 (IRE1) and eukaryotic translation initiation factor 2A (eIF2A) phosphorylation, and C/EBP homologous protein (CHOP) upregulation—a cascade activated via ER stress [62]. Of note, the phosphorylation of eIF2a results in the downregulation of global protein synthesis and in apoptosis through ER stress [63]. Overall, these data indicate that CHOP participates in fucoidan-induced DNA damage and suggest that this compound is able to modulate ER stress in breast cancer cells [27].

Later, Xue et al. [28] investigated the possible involvement of the phosphoinositide 3-kinase/protein kinase B/glycogen synthase kinase 3 beta (PI3K/PKB/GSK3b) pathway in the in vitro and in vivo actions of fucoidan [28]. Fucoidan treatment either in vivo (in DMBA-induced mammary cancer in rats, at 200 and 400 mg/kg, by oral route, for 16 weeks) or in vitro (in MDA-MB-231 cells, at 6.25–25 mg/mL) decreased the levels of p-PI3K, p-AKT, and p-GSK-3b (Ser9). The levels of β-catenin were also diminished [25]. Another suggested mechanism for fucoidan is the downregulation of β-catenin through the PI3K/AKT signaling pathway, which can regulate the phosphorylation of GSK3β, or by directly affecting β-catenin [28].

Interestingly, in rats with DMBA-induced mammary cancer, fucoidan (200 and 400 mg/kg, p.o., 4 months of treatment) also presented an immunomodulatory effect through the programmed death-1/programmed death-ligand-1 (PD1/PDL1) pathway. It was found that higher numbers of blood natural killer and CD4 and CD8 T cells, as well as the serum levels of interleukin (IL)-6, IL-12p40, and interferon (IFN)-γ, were elevated in rats treated with fucoidan. On the other hand, the percentage of Foxp3^+^ regulatory T cells and the levels of IL-10 and TGF-β were lower. A reduction of Foxp3^+^ was also found in the tumor tissue, together with a reduction of PDL-1, PIK3, and p-AKT and augmented INF-γ levels. These results suggest the immunomodulatory effect of fucoidan in DMBA-induced tumors is mediated by a PD1/PDL1/PI3K-AKT axis [29].

He et al. [30] evaluated the effects of the serum collected from healthy rats administered with fucoidan (MW: 675.5 kDa, 200 and 400 mg/kg, by oral route, for 3 days) on the growth of MCF-7 cells. The serum significantly suppressed MCF-7 cell proliferation, migration, and invasion, whilst enhancing apoptosis [30]. These effects were associated with up-regulation of E-cadherin and downregulation of matrix metalloproteinase-9 (MMP-9). In addition, the study also suggested that cell invasion and migration were possibly inhibited via the decreased epithelial–mesenchymal transition (EMT) process [30]. More recently, Hsu et al. [31], investigated the effects of the fucoidan isolated from the algae *Laminaria japonica* (34% of ester sulfate, MW: 80 kDa) in MDA-MB-231 cells. By using concentrations of 0, 0.125, 0.25, 0.5, 1, and 2 mg/mL (24 and 48 h of incubation), the authors found inhibition of the cell viability. The concentration of 2 mg/mL inhibited cell migration and invasion, suggesting that fucoidan inhibits tumor aggressiveness without inducing cell death. This effect was correlated with the downregulation of ERK, PI3K/AKT, mitogen-activated protein kinase (MAPK), c-Jun N-terminal kinase (JNK), mammalian target of rapamycin (mTOR), activator protein 1 (AP-1), and nuclear factor-kappa B (NF-κB) pathways. In addition, the antiangiogenic activity of fucoidan was confirmed in MDA-MB-231 cells through the reduction of VEGFA, IGF-I, MMP-2, and MMP-9 levels, basic fibroblast growth factor (bFGF), and the blockade of the adhesion and extravasation of tumor tissue to vascular endothelial cells. Fucoidan was also capable of inhibiting micro metastasis when MDA-MB-231-GFP cells were injected into the perivitelline cavity of zebrafish. Together, this set of data suggests that *Laminaria japonica* fucoidan has a potent antitumor effect by inhibiting metastasis and angiogenesis in triple-negative breast cancer cells [31].

Similarly, Zhang et al. [32], investigated the effect of a low molecular weight fucoidan (LMWF) (MW: 0.5 kDa) extracted from the brown algae *Cladosiphon novae-caledoniae* Kylin in breast cancer cells. This LMWF induced the apoptosis of MCF-7 and MDA-MB-231 cells when tested at the concentration of 820 ug/mL, an effect that was found to be time-dependent. The authors also demonstrated that LMWF-induced apoptosis of MCF-7 cells was caspase-independent and involved mitochondrial dysfunction, with alterations of Ca^2+^ homeostasis and reduction of the mitochondrial membrane potential, and the subsequent induction of cytochrome c release, in addition to a decreased expression of antiapoptotic Bcl-2 family proteins [32]. The activation of this mitochondrial apoptotic pathway was associated with reactive oxygen species (ROS)-dependent JNK phosphorylation. Although most of the mechanisms of action were observed for LMWF in MDA-MB-231 cells, they were found to be associated with caspase activation [33].

Another LMWF (MW: 0.5–0.8 kDa) from brown seaweed *Sargassum hemiphyllum* also exhibits antiproliferative, anticlonogenic, and anti-migratory effects in breast cancer cells (MCF-7 and MDA-MB-231 cells). In addition, the LMWF from brown seaweed when incubated for 48 h at 200 µg/mL caused up-regulation of miR-29c miRNA and downregulation of miR-17-5p miRNA expressions in MDA-MB-231 cells. The up-regulation of miR-29c was independent of the downregulation of miR-17-5p, associated with decreased levels of ADAM12 and TGFB, resulting in EMT and metastasis inhibition. On the other hand, the downregulation of miR-17-5p was associated with increased levels of PTEN and downregulation of PI3K and AKT phosphorylation and consequently inhibition of cancer cell survival. These findings indicate that the LMWF from the brown seaweed *Sargassum hemiphyllum*, inhibits breast cancer progression by regulating miR-29c/ADAM12 and miR-17-5p/PTEN axes [34].

Furthermore, another study found that the incubation of fucoidan (100 µg/mL for 48 h) silences β1-integrin gene expression and inhibits apoptosis in MCF-7 cells. Caspase 8 activation was also described. It suggests that β1-integrin plays an important role in the interaction between fucoidan and the cell surface. This interaction was indispensable for caspase 8 activation and apoptotic death of MCF-7 cells. Although the results are interesting, the study lacked detailed information on the source and molecular weight of fucoidan, impairing its full comparison with the above-mentioned studies [35].

Four additional studies were found. Two from the red algae *Laurencia papillosa* and 2 from brown algae *Undaria pinnatifida.* Murad et al. [36], demonstrated the antiproliferative effect of ι-Carrageenan in MDA-MB-231 cells, which was correlated with apoptosis through an extrinsic pathway [36]. It was later shown that the lowest tested concentration (10 µg/mL) of a sulfated polysaccharide (namely ASPE) from *Laurencia papillos* induced G1-phase arrest, which was accompanied by up-regulation of Cip1/p21 and Kip1/p27 and downregulation of cyclin D, cyclin E transcripts, and their related inhibitors Cdk2, Cdk4, and Cdk6 [64]. When incubated at a high concentration (50 µg/mL), ASPE triggered apoptosis in MDA-MB-231 cells through increased caspase-3 and Bax expression and decreased levels of Bcl-2. In addition, over-generation of ROS was associated with ASPE-induced apoptosis. However, it was unclear on whether ASPE consists of ι-carrageenan. Afterward, the effects of κ-, ι-, and λ-carrageenan, namely, LP-W1, LPW-2, and LPW-3, respectively, were investigated in MCF-7 cells [64]. Interestingly, only LPW-2 and LP-W3 (at 50, 100, 150, and 200 µM for 24 h) significantly inhibited cell proliferation, activated apoptotic genes such as PARP, caspase 3, and p53, and reduced Bcl-2 levels [37].

A polysaccharide from the brown algae *Undaria pinnitafida* was also studied. The authors conducted a study evaluating the effect of a sulphated polysaccharide from *U. pinnitafida* (SPUP) on DMBA-induced mammary cancer in rats. SPUP (300 mg/kg, by oral route, for 20 weeks) significantly inhibited tumor growth. The study described an immunomodulatory activity supported by the increased organ index (spleen and thymus). In addition, sex hormones such as estradiol, progesterone, prolactin, luteinizing hormone, and follicle-stimulating hormone, all imbalanced in DMBA vehicle-treated animals, were restored by SPUP administration. The result suggests that the anticancer mechanism of SPUP may be related to hormonal sex regulation. Later, the same group showed the antitumor effect of SPUP in MCF-7 cells. SPUP (25, 100, and 200 µg/mL) decreased cell viability in a concentration- and time-dependent manner. In addition, SPUP inhibited colony formation and migration and induced cell apoptosis [39].

### 3.6. Bacteria

Queiroz et al. [40] worked with MCF-7 cells and studied the effects of a levan (2→6)-β-D-Fructan polysaccharide. Levan is a homopolymer of fructose units linked mainly by β-(2→6)-D-fructofuranosyl bonds, with occasional β-(2→1)-linked branched chains. It is produced in the extracellular matrix by a wide range of bacterial species [39]. The study isolated the levan polysaccharide from the gram-negative bacteria *Halomonas smyrnensis* AAD6T. This polysaccharide presented time (24 and 48 h)- and concentration (0–1500 µg/mL)-dependent antiproliferative activity. Interestingly, levan caused apoptosis by increasing oxidative stress and inducing p53 (TP53 gene) and p27 (CDKN1B gene) gene expression (100 µg/mL for 48 h). The percentage of apoptotic cells in the sub-G1 phase was increased by this polysaccharide, whilst the percentage of those in G0-G1 phase was decreased. An increase in caspase 3/7 activity was also observed. Therefore, levan polysaccharide exhibited an antiproliferative effect in MCF-7 cells that was mediated by an increase in apoptosis and oxidative stress [40].

### 3.7. Plants

There were five studies with plants, including two that employed in vitro and in vivo techniques and three studies that performed only in vitro experiments. Interestingly, the majority of the studies were from China, a well-known country for the study of herbal products.

Among the traditional Chinese medicine, Ruyiping has gained attention as a medicinal formula for preventing postoperative recurrence and metastasis of breast cancer patients. Ruyiping is composed of *Pseudobulbus cremastra* seu pleiones (Shancigu), *Nidus vespae* (Lufengfang), *Curcuma zedoaria* (ezhu), raw seeds of *Coix lacryma-jobi* L. var. mayuen (Roman.) Stapf (Shengyiyiren), and *Akebiae fructus* (Bayuezha). This herbal medicine (at 40%) inhibits the growth and invasion of breast cancer by inducing cell cycle arrest and reducing MMP9 and EMT [41]. Ruyiping formula active components include *Pseudobulbus cremastra* seu pleiones polysaccharide (PCSPP) and curcumol. Indeed, PCSPP and curcumol were found to inhibit cell proliferation and EMT-related markers, suggesting that both components are responsible for Ruyiping effects. However, a limitation of the study is that no other component of Ruyiping formula was investigated [41].

Another Chinese herbal well explored is *Astragalus membranaceus*, which has been used for centuries as an immune stimulator, antiviral, antioxidant, and antitumor agent [65,66,67,68,69,70]. Astragalus polysaccharide (APS) was identified as one of the bioactive chemicals present in *Astragalus membranaceus* [66]. However, little is known of the mechanisms of action of APS in breast cancer. Thus, Liu at al. [42] employed an in silico method based on network pharmacology to screen APS mechanisms. Then, APS effects were investigated in MCF-7 and MDA-MB-231 cells. MCF-7 proliferation was significantly decreased after APS incubation (0.25–2 mg/mL; for 96 h), whereas in MDA-MB-231 cells, only the highest concentrations (0.75–2 mg/mL) inhibited proliferation. Migration was also inhibited by ASP (0.25–0.5 mg/mL) in both tested cell lineages, and the expression of CCNB1 and CDC6 was decreased. p53 expression was increased in MDA-MB-231 cells. The results suggest a therapeutic potential for APS in breast cancer, possibly through modulation of CCNB1, CDC6, and p53 [42].

The roots of Yulangsan (*Milleta pulchra* Kurz var-laxior (Dunn) Z.Wei) are also famous herbal medicine in China with several biological properties, such as anti-inflammatory, anti-fibrosis, anti-viral, enhanced immunity, and antitumor activities [71,72,73]. Yulangsan polysaccharide (YLSPS) is the major effective extract from Yulangsan roots, and its antitumor effect has attracted considerable attention [43]. Therefore, Qin et al. [43] examined the antitumor effect of YLSPS on breast cancer cells. YLSPS was given to Sprague-Dawley rats at the doses of 750, 1500, and 3000 mg/kg by oral route, twice a day for 7 days. The blood was collected, and the serum of the animals was cultured in RPMI medium to be further incubated with 4T1 cells as conditioned serum [74]. All tested concentrations of YLSPS decreased cell viability and increased apoptosis by increasing Bax and caspase-3 expression and reducing Bcl-2 levels. The in vivo transplantable tumor model (BALB/c mice injected with 4T1 cells) showed that YLSPS inhibited tumor growth from day 7 to day 14 after tumor inoculation in YLSPS-treated rats (150–600 mg/kg). A reduction of serum MDA and increased SOD and GPX levels were observed in these animals, indicating an antioxidant action for YLSP. Apoptosis and reductions of VEGF and angiogenesis levels were observed in the tumor’s samples treated with YLSP [74].

In another study, the dried tuber of *Sparganium stoloniferum* Buch. -Ham. (Sparganii Rhizoma), a traditional Chinese herbal medicine frequently used in the treatment of hyperacidity and gynecology disorders and in oncology (Pharmacopoeia of People’s Republic of China, part I, 2015 edition), was investigated [44]. As the Sparganii rhizoma contains a substantial amount of polysaccharide, Wu et al. [44] isolated one from it, named SpaTA, by water extraction. The structure of SpaTA was described as a backbone consisting of 2-Ograilsine-β-xylose (4→6)-α-glucose (1→4) -β-mannose osamine [44]. An aluminium element combined with nitrogen on both grailsine and mannose osamine was identified in the repeating unit of SpaTA. The molecular weight was 5.250 kDa, and the monosaccharide composition consisted of xylose, glucose, and mannone osamine. ZR-75-1 (breast cancer cells ERα positive) was only at the concentration of 611.2 mg/L and inhibited cell proliferation after 72 and 96 h incubation [44]. The same concentrations, as well as intermediate ones (152.8, 305.6 mg/L) of ZR-75-1, inhibited cell growth at 24 and 48 h. SpaTA (611.2 mg/L) also increased the percentage of S-phase cells within 48 h. Additionally, increased numbers of apoptotic cells and necrotic cells were observed between 72–96 h [44]. The results indicate that the incubation time is an important factor. At 305.6 and 611.2 mg/L, SpaTA also induced ERα expression in ZR-75-1 cells at nuclear level, with concomitant increased levels of caspase 3, 8, 9, and PARP at 72 h (these results were not observed in cells knocked down for ERα). The increased apoptotic proteins were confirmed to be ERα-dependent, since in MDA-MB-231 cells (triple negative breast cancer cells), this effect was not observed [44].

*Pyracantha fortuneana* (Maxim.) Li (Huo-ji) is distributed mainly in the northwest of China, and its fruits are known to be enriched with Selenium (Se). The polysaccharides of *P. fortuneana* (PFPs) were previously shown to suppress inflammation and oxidative stress and to be hepatoprotective, antitumor, and antiviral [75,76,77,78]. Therefore, Yuan et al. [45] investigated whether polysaccharides extracted from Se-enriched *P. fortuneana* (Se-PFPs) presented anticancer activity in breast cancer cells [58]. The extract contained 93.7% (*w*/*w*) of carbohydrate, 2.1% of uronic acid, and 3.7 ug/mL of Se. The heteropolysaccharide was composed of xylose, arabinose, fucose, mannose, ribose, rhamnose, glucuronic acid, galacturonic acid, glucose, and galactose. In triple-negative breast cancer cells (MDA-MB-231) Se-PFPs (up to 400 μg/mL) inhibited cell growth in a concentration-dependent manner at 24 and 48 h following incubation. This effect was associated with cell cycle arrest at the G2 phase (400 ug/mL of Se-PFPs after 24 h). The cell cycle arrest was suggested to be via inhibition of the cyclin B1/CDC2 pathway, enhancement of apoptotic proteins, such as caspase 3/9, p53, Bax, Puma, and Noxa, and decreased Bcl-2 expression. Treatment of nude mice bearing MDA-MB-231-derived xenograft tumors with Se-PFPs (100 and 400 mg/kg, by oral route, for 30 days) significantly reduced tumor growth [45]. However, the in vivo mechanism of this polysaccharide was not investigated. Overall, Se-containing polysaccharides extracted from *P. fortuneana* presented an antitumor effect in breast cancer cells, probably through apoptosis induction, indicating its potential to treat triple-negative breast cancer [45].

### 3.8. Fruits

Five studies that explored polysaccharides isolated from fruits were found. Three studies used in vitro methodologies only, while two studies used in vitro and in vivo methodologies.

Two studies evaluated the effects of polysaccharides isolated from the *Lycium barbarum* (Wolfberry, commercially known as Gojiberry). The chemical characterization of wolfberry-derived polysaccharides (namely WFPs) revealed they were soluble in water and were non-starch protein-bounded acidic polysaccharides [46]. WFPs were mainly composed of D-galacturonic followed by D-galactose, L-arabinose, and D-glucose. The experiments designed with MCF-7 cells showed that WFPs (25–200 µg/mL for 24 and 48h) presented antiproliferative effects and at the highest concentration (100 and 200 µg/mL), induced cell cycle arrest at G0/G1. Moreover, flow cytometry analysis showed that WFPs exert a stimulatory effect on apoptosis and induce ROS production and DNA damage in a concentration-dependent manner. In conclusion, WFPs exert anticancer activities mediated by induction of apoptosis and ROS damage, which results in an antiproliferative effect [46].

Huang et al. [47] aimed to elucidate the transduction pathways responsible for mediating breast-cancer-suppressive effects of the polysaccharides isolated from *Lycium barbarum* (namely in the study as LBPs; MW: 30–100 kDa). By using MCF-7 cells, they found that 0.50 mg/mL of LBPs inhibits cell proliferation in a time-dependent manner and is antiangiogenic by inhibiting insulin-like growth factor (IGF)-1 protein and hypoxia-inducible factor-1 (HIF-1). The inhibition of IGF-1 was associated with the suppression of phosphatidylinositol 3-kinase (PI3K) activity, phosphorylated-PI3K (p-PI3K), and VEGF levels [47].

The polysaccharides from Hallabong peels were studied by Park et al. [48]. Hallabong is a type of orange from the *Citrus sphaerocarpa* found mainly in South Korea. Four polysaccharides were extracted from the peels, namely HB-I (MW: 64 and 60 kDa), HB-II (MW: 25kDa), HB-III (MW: 10 and 5.3 kDa), and HB-IV (MW: 4kDa). In MDA-MB-231 cells, only the highest tested concentration (100 µg/mL) of HB-II inhibited cell proliferation. No differences were found in angiogenesis markers following HB-II incubation. However, a reduction of MMP-9 expression was observed after HB-II treatment (25 µg/mL), suggesting its potential antimetastatic effect. Nonetheless, the results are preliminary, and additional investigations should be performed to further evaluate this effect [48].

Delphi and Sepehri [49] studied a pectic acid polysaccharide isolated from an apple. This polysaccharide has been demonstrated to have important effects on cancer pathways. The authors showed that an apple pectic acid polysaccharide (0.1–1%) inhibited cell growth, induced apoptosis and decreased cell attachment, fragmented chromatin, and membrane blebbing. It also blocked the sub-G1 phase in 4T1 cancer cells. In a complementary way, by using female BALB/c mice, they demonstrated that daily supplementation with 1% of pectic acid polysaccharide for 3 weeks inhibited tumor growth and metastasis via over-expression of p53 and a high rate of apoptosis in tumor tissue [49].

Adami et al. [50] also studied a pectic polysaccharide. The polysaccharide fraction was obtained from the green sweet pepper (*Capsicum annuum*), and named CAP. The fraction was mainly composed of uronic acids, with minor amounts of rhamnose, arabinose, xylose, galactose, and glucose. CAP consisted of a highly methoxylated homogalacturonan with a type I arabinogalactan anchored to rhamnogalacturonan. CAP (100 mg/kg, by oral route, for 21 days) reduced Ehrlich tumor growth in vivo, and at the concentration of 0.1 mg/mL, it decreased the viability and colony formation of MCF-7 and MDA-MB-436 human mammary cells. These results were associated with reduced gene expression of VEGF in vivo and in vitro. The polysaccharide fraction also reduced vessel areas, promoted necrosis, and increased IL-6 in the tumor tissue [50].

### 3.9. Fungus

Four studies with polysaccharides isolated from fungus (not mushrooms) were found. Only one study included animal experiments. Two studies evaluated the polysaccharide β-D-glucan. Jafaar et al. [51], studied a β-1-3-D-glucan isolated from *S. cerevisae* and compared its effects in endocrine-sensitive MCF-7 versus endocrine-resistant LCC9 and LY2 breast cancer cell lines. The cells were exposed to different concentrations β-glucan (1–400 μg/mL) for 48 and 72 h. The β-D-glucan inhibited cell proliferation and increased Bax/Bcl-2 ratio in a concentration-dependent manner. On the other hand, the inhibition of NRF1 did not appear to be dependent on the concentration. The authors also showed regulation of the breast cancer-relevant gene expression (RASSF1 in MCF-7 cells; IGFBP3, CTNNB1, and ERβ in LCC9 cells) with β-D-glucan at 10 and 50 µg/mL, indicating that this compound represents an attractive option for inhibiting endocrine-resistant breast cancer cell proliferation [51].

Another study evaluated the effects of three fungal exocellular β-glucans. The β-glucans were obtained from *Botryosphaeria rhodian* MAMB-05 (two botryosphaerans; (1-3)(1-6)-β-D-glucan; one produced from glucose, the other from fructose) and *Lasiodiplodia theobromae* MMPI (lasiodiplodan; (1-6)- β-D-glucan, produced from glucose) in MCF-7 cells. Botryosphaeran from glucose exhibited a time- and concentration-dependent antiproliferative activity, with a half-maximal inhibitory concentration of 100 μg/mL. Botryosphaeran from fructose presented the best antiproliferative response at 1500 µg/mL. The results with lasiodiplodan were similar to botryosphaeran. All the β-glucans increased oxidative stress, apoptosis (through induction of p53 and Bax), and necrosis in MCF-7 cells. Interestingly, the apoptosis induced by β-glucans (100 µg/mL for 48 h) was found to be mediated by AMP-activated protein-kinase and forkhead transcription factor FOXO3a [52].

Wang et al. [53], studied an exopolysaccharide (named EPS) isolated from the fermentation liquor of *Trichoderma pseudokoningii* fungus. The incubation of MCF-7 cells with EPS at 0.10, 0.25, 0.50, 0.75, and 1.0 mg/mL for 24, 48, and 72 h decreased cell viability and increased LDH release in a concentration-dependent manner. Apoptosis induction through activation of caspase-3, caspase-8, and caspase-9 and increased Bax/Bcl-2 ratio was described at 0.25, 0.50, and 1 mg/mL. Furthermore, EPS promoted the release of cytochrome c into the cytoplasm and disruption of mitochondrial membrane potential. Thus, EPS induced the apoptosis of MCF-7 cells through an intrinsic mitochondrial apoptotic pathway and may therefore be considered an effective adjuvant agent against human breast cancer [53].

The only study that included animal experimentation was conducted by Xie et al. [54]. In very well-designed study, they studied a non-ionic, non-immunogenic polysaccharide produced by *Aureobasidium pullulan*, a spermine modified pullulan (PS, MW: 90.000). Focusing on the immunomodulatory function, they first demonstrated in vitro, using Raw 264.7 cells, that the pullulan polysaccharide upregulated the expression of toll-like receptor 1/3 and 4 and promoted the phosphorylation of AKT, ERK, and JNK and the activation of the NF-κB pathway. They suggested that this response may be related to M1 polarization. By using a heterotopic breast cancer model (4T1 in vivo cell inoculation), the polysaccharide (2.4 mg/kg, by the subcutaneous route, 11 times for 21 days) induced an effective antitumor effect through reprogramming of M2 to M1 in the tumor microenvironment. Increased CD4^+^ and CD8^+^ T cells and decreased expression of CD31 in the tumor mass were also observed [54].

### 3.10. Mushrooms

Gan et al. [55] designed a study to show the antiproliferative and apoptotic activities of an acidic polysaccharide fraction from *Pholiota dinghuensis Bi* mycelium, named PDP-3 (MW: 353 kDa), using MDA-MB-231 and MCF-7 cells. PDP-3 contained high contents of uronic acid, protein, and sulphur radical. The sugar composition of PDP-3 contains glucose, mannose, xylose, galactose, fructose, and rhamnose. PDP-3 prevented cell growth (EC50 = 38.35 µg/mL) in both MDA-MB-231 and MCF-7 cells in a concentration-dependent manner. Proliferation inhibition in MCF-7 cells (PDP-3, 13–52 µg/mL) was associated with diminished proliferating cell nuclear antigen (PCNA), cyclin D1, and cyclin-dependent kinase 4 (CDK4) expression. Furthermore, PDP-3 also induced apoptosis through the stimulation of p21, caspase 3, and Bax expression and downregulation of Bcl-2 and caspase-9. Downregulation of TNF receptor-associated factor 2 (TRAF2) and up-regulation of p38, p53, and apoptosis signal-regulating kinase 1 (ASK1) phosphorylation were also described. In addition, PDP-3 decreased both the nuclear and cytoplasmic expression of ERα. In summary, PDP-3 inhibited tumor proliferation and induced apoptosis through the activation of p38/MAPK [55]. It is important to point out that PDP-3 is also able to downregulate ER in MCF-7 cells, contributing to its antitumor effect.

Thus, Luo et al. [56] evaluated the polysaccharides (namely as SP1) present in Hauier fruiting bodies in breast cancer cells. The total carbohydrate content of SP1 was ~94%, and no uronic acid or proteins were detected. The monosaccharide composition included galactose, arabinose, and glucose, and the molecular weight was 56 kDa. SP1 inhibited MCF-7, MDA-MB-231, and MDA-MB-435 cell proliferation in concentrations higher than 400 µg/mL. In MCF-7 cells, SP-1 (at 100, 200, and 400 µg/mL) induced apoptosis through increasing Bax expression and decreasing MTDH levels, which is frequently upregulated in various human tumor types, including breast cancer [56].

*Cordyceps* sp. a parasitic type of mushroom, is also a traditional Chinese medicine and known to contain large amounts of polysaccharides. Lin et al. [57] studied a newly isolated polysaccharide (named MHP-1) from *Mortierella hepialid,* the asexual structure of *Cordyceps sinensis*. In vitro, MHP-1 (0.1–1 µM) decreased cell migration in MDA-MB-231, MCF-7, and MDA-MB-468 cells. In these cells, E-cadherin and zonula occludens-1 (ZO-1) expressions were increased, and vimentin and fibronectin levels were diminished. The transcription factor snail was also significantly downregulated. MHP-1 also inhibited the protein levels of slug, suppressed EMT breast cancer, and decreased MMP-9 secretion. Furthermore, TGF-β signaling was inhibited and the sensitivity in topotecan (TPT)-resistant MCF-7 cells was restored. By using a xenograft model in nude mice (MDA-MB-231) (60 mg/kg, by the intravenous route, for 4 weeks), the authors showed MHP-1 treatment led to inhibition of breast cancer metastasis, followed by a decreased expression of TGFβ type I receptor kinase (ALK5) and vimentin and increased expression of E-cadherin by MHP-1 [57].

The other two studies evaluated lentinan (LTN), a β-(1→6) branched β-(1→3)-glucan derived from the mushroom of *Lentinus edodes*. Xu, Zou, and Xu. [52], studied LTN in ER^+^ human breast cancer cell lines (MCF-7 and T47D) and triple-negative cell lines (MDA-MB-231 and MDA-MB-468) [58]. Interestingly, only the proliferation of MCF-7 and T47D cells was inhibited by LTN (12.5–400 µg/mL). LTN targeted p53 and ERα to inhibit MCF-7 cell proliferation. Additionally, 1 mg/kg LTN decreased tumor growth in female athymic BALB/c nude mice (inoculated with MCF-7 cells). In addition, apoptosis was observed in the tumor tissue; this was associated with increased p53 and caspase 3 and reduction of Bcl-2 expression. The suppression of cell proliferation and apoptosis is probably through multiple pathways such as p53-dependent, caspase, PI3K/AKT/mTOR, NF-κB-, ERK-, and ERα pathways. Overall, this study provided new insights to treat ER^+^ breast cancers using LTN [58].

Similarly, Li Z et al. [59] investigated in more detail the anticancer mechanism of LTN in MCF-7. An aqueous LTN extract (ALNT) was prepared from *Lentinus edodes*. The sugar content was ~96% and the molecular weight was 630 kDa. ALNT (250–1000 µg/mL) modulated autophagy by increasing LC3 conversion and decreasing p62. Apoptosis was also observed and was related to increased caspase 7 and Bax expression in MCF-7 cells. An antitumor effect of ALNT (5–20 mg/kg, i.v. for 3 weeks) was also observed in a xenograft in vivo model using MCF-7 cells. ALNT inhibited tumor growth, apoptosis, and autophagy. An increased expression of caspase 7 and Bax was observed in vivo, in addition to cytochrome c release and LC3 conversion [59].

## 4. Discussion

**Figure 4:**
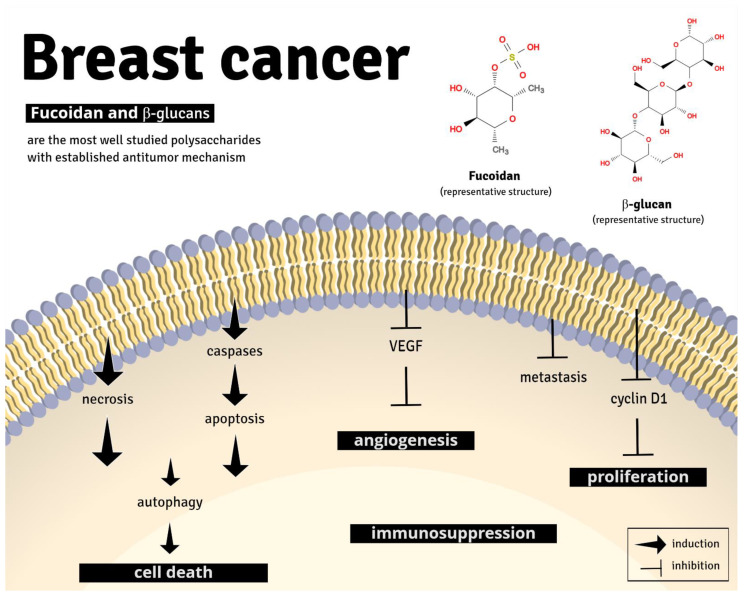
Representative image of the main anti-tumor pharmacological mechanism of fucoidan and β-glucans. Fucoidan and β-glucans can induce apoptosis, cell death, and immune suppression and inhibit angiogenesis, metastasis, and cell proliferation, through different signaling pathways (described in detail in Table 1).

Analysis of non-clinical breast cancer models revealed that the mechanisms of action of polysaccharides involve apoptosis, inhibition of cellular proliferation, and angiogenesis, in addition to antimetastatic effects through multiple pathways. Clearly, fucoidan and β-glucans are the most well-studied polysaccharides with established antitumor mechanism (Figure 4).

Fucoidan, also called fucan or fucoidan, is a sulfated polysaccharide mainly found in different species of brown algae, as part of their cell walls [79]. Among the brown algae species containing fucoidan, *Fucus vesiculosus* was the most investigated. The studies indicate that the structures and compositions of fucoidan vary among brown algae species; however, they generally contain a black bone of sulfated fucan, along with small quantities of D-galactose, D-mannose, D-xylose, and uronic acid. Fucoidan has been extensively studied since its first isolation in 1913 and has been consumed as a dietary fiber in many Asian countries [24,80]. Fucoidan presents a variety of biological activities, including antithrombotic, antiviral, antioxidant, anti-inflammatory, and immunomodulatory actions. Its anticancer effects have gained considerable attention due to its antitumor properties in B- and T-cell lymphoma, leukemia, fibroblastic and uterine sarcoma, osteosarcoma, hepatocellular and endometrium carcinoma, melanoma, and lung, colorectal, bladder, stomach, pancreatic, ovarian, and prostate cancer [80]. In breast cancer models, fucoidan induced apoptosis and inhibited cell proliferation through interaction with several signaling pathways [35]. Importantly, fucoidan reduced tumor growth and weight and metastasis [26,60]. Some inconsistencies were found between the studies [26,27], concerning the dose used to investigate the anti-tumor effect of fucoidan. For instance, the dose used by Hsu et al. [31], was higher compared to the study conducted by Chen et al. [27].

This particularity also reflects in the results found by Zhang et al. [32] that studied a LMWF from the algae *Laminaria japonica* [32]. The molecular weight is generally classified as low (≤10 kDa), medium (10–10.000 kDa), and high (≥10.000 kDa) [81], and it is known that LMWF is more soluble in water; thus, a higher bioavailability in vivo was observed [32]. Therefore, the differences may occur because different types of extraction (chemical, physical, or enzymatic treatment) and purification methods to obtain fucoidan can result in a distinct structure and molecular weight, and consequently, different pharmacological effects.

The anticancer effect of fucoidan is also well consolidated in animal models. Importantly, one study showed the potential immunomodulatory effect of fucoidan on breast cancer cells through the de PDL-1 axis, and all the effects were observed in triple-negative breast cancer cells (MDA-MB-231 and 4T1 cells) or estrogen-sensitive cells (MCF-7).

Both fucoidan from *Fucus vesiculosus* and *Undaria pinnatifida* are approved in the USA by Food and Drug Administration (FDA) in the Generally Recognized As Safe (GRAS) category as food ingredients, based on the lack of toxicity in vivo and in a clinical trial [82,83]. Furthermore, several studies explored the anticancer potential effect of fucoidan in different types of cancer as an adjuvant treatment and its ability to improve patient’s quality of life [84]. However, although it has been well explored, no clinical studies have been found with fucoidan in regarding to breast cancer.

β-glucans have also been extensively studied and have attracted considerable attention worldwide. Glucans refer to a diverse class of glucose polymers that can be short or long, branched or unbranched, α or β isomers, and soluble or particulate. Particularly, β-1,3-D-glucans can be found in yeast (typically *Saccharomyces cerevisiae*), mushrooms, bacteria, plant cell walls, and seaweed. β-1,3-glucan has important interactions with the human body, mainly with the immune system, which gives this type of polysaccharide a wide range of biological effects [85]. β-glucans can exhibit direct and indirect antiproliferative effects against breast cancer cells, and the findings provide valuable information about the possible mechanistic insights about its antiproliferative role [83]. Among β-glucans, the best-investigated polysaccharide was Lentinan. Lentinan is described to have an anticancer effect on gastric, hepatic, ovarian, and lung cancer. Lentinan was studied in human breast cancer cell lines of ER^+^ (MCF-7 and T47D) and triple-negative cell lines (MDA-MB-231 and MDA-MB-468) and inhibited only MCF-7 and T47D cell proliferation.

Of importance, β-glucans are already employed as adjuvant therapy in cancer treatment to improve the immune system response [83]. Several clinical trials already reported increased quality of life and an immune stimulatory effect of β-glucans in patients with breast cancer under chemotherapy. Increased blood cells count, serum levels of IL-4 and IL-12 and activation of peripheral blood monocytes were described [52,86,87,88]. However, none of the studies selected in our systematic review evaluated the immunostimulatory effect of β-glucans per se in breast cancer models. β-glucans from yeast are also approved in the USA by FDA as GRAS [82,89], but no clinical trials were performed yet on breast cancer patients.

Other polysaccharides described here may also be candidate agents for more detailed studies and perhaps clinical trials. The Levan polysaccharide derived from bacteria and the polysaccharides from red and brown algae (*Laurencia papillosa* and *Undaria pinnatifida*), as well as some pectins from fruits, showed an interesting antitumor response [38,39]. At least two studies documented the antitumor effect. However, in vivo experiments, well-structured in relation to dose and treatment time, and those that aim to assess toxicity with well-designed methodologies and correct statistical tests should be performed.

For instance, Delphi and Sepehri [49] and Adami et al. [50] arranged their studies including in vivo and in vitro methodologies to study pectic polysaccharide isolates from fruits. This strategy brings up advanced and robust information to better comprehend the polysaccharide activity and mechanism of action [49,50].

Certainly, other polysaccharides included in this systematic review also attracted attention for their initial results. However, those studies are still in the beginning, and more detailed investigations need to be conducted to engender a comparative discussion. Only a few studies attempted to investigate the immunomodulatory activity of polysaccharides. None of the articles investigate the HER2 or progesterone receptor involvement with the polysaccharide antitumor effect, and only three evaluate the estrogen receptor. Particularly, these experiments should be included when evaluating the anticancer effect of molecules to provide knowledge of which type of breast cancer the molecule could be used to treat. Importantly, most of the studies only employed in vitro experiments. The inclusion of in vivo animal models is strongly encouraged. Xenograft models, using human breast cancer cells, are closer to human conditions and could provide more information on the effect of these molecules as well as the potential toxicity. Furthermore, although studies make little or no inference, the relation between the structural characteristics and pharmacological activity of the polysaccharide must be considered to facilitate subsequent studies.

Finally, we discussed here only articles with high-quality methodology and low risk of bias. Additional studies are required to elucidate the polysaccharides’ effects, mechanism of action, and toxicity before the initiation of translational research and clinical studies. Our systematic review also provides new insights to improve the knowledge of which polysaccharides could be further investigated for the treatment of breast cancer.

## 5. Conclusions

We performed a systematic review of non-clinical studies reporting on the antitumor effect of naturally occurring polysaccharides and their mechanism of action. A thorough search was carried out to include only studies with high quality of evidence and without risk of bias. Parallel considerations of the polysaccharides that were better explored and with a well-defined mechanism of action in non-clinical trials have enabled an exploration of the extent to which one may have the potential for translational research and clinical studies on breast tumor. There appears to be strong evidence in support of some polysaccharides’ ability as new intervention strategies in the treatment of breast cancer. However, there is still a deficiency regarding data concerning structural polysaccharides’ characteristics, bioavailability, and pharmacokinetics. Fucoidan and β-glucan polysaccharides appear to be the most well-defined polysaccharides concerning their mechanism of action in non-clinical models. Translational research into clinics as monotherapy still needs to be strategically evaluated before it becomes a reality in the field of cancer control.

## Figures and Tables

**Table 1:** Description of the PICO (Participants, Intervention, Comparison, and Outcome) criteria used in the present systematic review.

CRITERIA	DESCRIPTION
PARTICIPANTS	Non-clinical studies referring to the biological activities of extracts or isolated polysaccharides in breast cancer models (e.g., female murine models of breast cancer and breast cancer cells)
INTERVENTION	Extracts or isolated polysaccharides treatment
COMPARISON(S)	Comparison control groups (which were not treated with polysaccharide)
OUTCOMES (S)	Changes in parameters related to the model of breast cancer studied

**Table 2:** Mechanism of action of different polysaccharides.

SOURCE	Species	Polysaccharide	In Vitro Model	In Vivo Model	Concentration/ Dose	Mechanism of Action/ Antitumor Effect	Reference
SEA ANIMALS	*Asterina pectinifera*	Ps	MCF-7	-	10, 20, 40 *, 80 *, 120 * µg/mL	Inhibit metastasis through COX-2 and MMP-9 via downregulation of MAPK pathway	Lin, Shi, and Nam (2013) [23]
ALGAES	*Fucus vesiculosus*	Fucoidan	4T1	4T1 (mice)	50 *, 100 *, and 200 * µg/mL 5 * and 10 * mg/kg	Apoptosis through mitochondrial pathway, inhibit angiogenesis through downregulation of VEGF and ERK signaling and inhibit metastasis	Xue et al. (2012) [24]
			4T1	4T1 (mice)	25 *, 50 *, 100 *, and 200 * µg/mL 5 * and 10 * mg/kg	Inhibit proliferation through downregulation of cyclin d1 and wnt/β-catenin signaling	Xue et al. (2013) [25]
			4T1 MDA-MB-231	4T1 (mice)	60, 90 *, 100 *, and 120 * µg/mL 0.25 * mg/kg	Inhibit metastasis through tgfr/smad/snail, slug, twist and emt axes	Hsu et al. (2013) [26]
			MDA-MB-231	-	10, 50 *, and 100 * µg/mL	Apoptosis through er stress	Chen et al. (2014) [27]
			MDA-MB-231	DMBA (rat)	6.25 *, 12.5 *, and 25 * µg/mL 200 * and 400 * mg/kg	Inhibit proliferation and induce apoptosis through downregulation of PI3K/AKT/gsk3β pathway	Xue et al. (2017a) [28]
			-	DMBA (rats)	200 * and 400 * mg/kg	Immunosuppression of treg through PD1/PDL1 pathway	Xue et al. (2017b) [29]
			MCF-7 with ex vivo sample (blood from Sprague Dawley Rats)	-	200 * and 400 * mg/kg	Inhibit proliferation, induce apoptosis, and inhibit migration through downregulation of MMP-9 and upregulation of e-cadherin	He et al. (2019) [30]
	*Laminaria japonica*	Fucoidan	MDA-MB-231	-	0.125, 0.25, 0.5, 1 *, and 2 * mg/mL	Inhibit angiogenesis and proliferation through activation of MAPK and PI3K followed by inhibition of ap-1 and NF-κB signaling	Hsu et al. (2020) [31]
	*Cladosiphon novae-caledoniae*	Fucoidan (LMWF)	MCF-7 MDA-MB-231	-	82, 410, and 820 * µg/mL	Apoptosis via mitochondria pathway associated with ROS-dependent JNK phosphorylation	Zhang et al. (2011) [32]
	*Cladosiphon novae-caledoniae*	Fucoidan (LMWF)	MDA-MB-231	-	200 * and 400 * µg/mL	Apoptosis through caspase activation and mitochondrial dysfunction	Zhang et al. (2013) [33]
	*Sargassum hemiphyllum*	Fucoidan (LMWF)	MCF-7 MDA-MB-231	-	50, 100, and 200 * µg/mL	Inhibit tumor progression through mir-29c/adam12 and mir-17-5p/PTEN axes	Wu et al. (2016) [34]
	-	Fucoidan	MCF-7	-	100 * µg/mL	Apoptosis through β1-integrin-caspase-8 complex	Yamasaki et al. (2012) [35]
	*Laurencia papillosa*	Aspe	MDA-MB-231	-	10 *, 50 *, and 100 * µg/mL	Inhibit proliferation through g1-phase arrest and induce apoptosis through caspase activation	Murad et al. (2015) [36]
	*Laurencia papillosa*	Carrageenan	MCF-7	-	25, 50 *, 100 *, 150 *, and 200 * µg/mL	Inhibit proliferation and induce apoptosis	Ghannam et al. (2018) [37]
	*Undaria pinnatifida*	Spup	-	DMBA (rats)	100, 200, and 300 * mg/kg	Reduce tumor growth, have immunomodulatory activity, and modulate sex hormones	Han et al. (2016) [38]
	*Undaria pinnatifida*	Spup	MCF-7	-	25 *, 100 *, and 200 * µg/mL	Inhibit migration and proliferation and induce apoptosis	Wu et al. (2019) [39]
BACTERIA	*Halomonas smyrnensis* aad6	Levan	MCF-7	-	10, 25, 50, 75, 100 *, 250, 500, 750, 1000, and 1500 µg/mL	Inhibit proliferation, induce apoptosis and oxidative stress	Queiroz et al. (2017) [40]
PLANTS	Ruyiping	Pcspp	MDA-MB-231 MDA-MB-468	-	1, 2.5, 5, 10, 20, 30, 40 *, 45, and 50%	Inhibit proliferation and emt-marker	Li et al. (2019) [41]
	*Astragalus membranaceus*	APS	MCF-7 MDA-MB-231	-	0.25 *, 0.5 *, 0.75, 1, and 2 mg/mL	Inhibit proliferation through inhibition of CCNDB1, CDC6, and p53	Liu et al. (2019) [42]
	*Millettia pulchra* Kurz var.	Yulangsan	4T1 with ex vivo samples (Blood from Sprague Dawley Rats)	4T1 (mice)	750 *, 1500 *, and 3000 * mg/kg 150 *, 300 *, and 600 * mg/kg	Inhibit angiogenesis through inhibition of VEGF, induce apoptosis through caspase activation and inhibit metastasis	Qin et al. (2019) [43]
	*Sparganii Rhizoma*	SpaTA	ZR-75-1 MDA-MB-231	-	76.4, 152.8, 305.6 *, and 611.2 * mg/L	Induce apoptosis through regulating ERα	Wu, Sun, and Wang (2017) [44]
	*Pyracantha fortunean*	Se-PFPs	MDA-MB-231	MDA-MB-231 (mice)	50, 100 *, 200, and 400 * µg/mL 100 * and 400 *, mg/kg	Inhibit proliferation by arresting cells at G2 phase via inhibiting CDC25C-CyclinB1/CDC2 pathway and induce apoptosis through p53-mediated cytochrome c-caspase pathway	Yuan et al. (2016) [45]
FRUITS	*Lycium barbarum* (wolfberry)	WFP	MCF-7	-	25, 50, 100 *, and 200 * µg/mL	Induce apoptosis and oxidative stress and inhibit proliferation through the g0/g1 cell cycle arrest	He et al. (2012) [46]
	*Lycium barbarum* (wolberry)	LBP	MCF-7	-	0.05, 0.1, 0.25, 0.5 *, and 1 mg/mL	Inhibit angiogenesis through IGF-1 and PI3K/HIF-1A/VEGF pathway	Huang et al. (2011) [47]
	*Citrus sphaerocarpa* (hallabong peels)	Hbe-ii	MDA-MB-231	-	1.56, 3.12, 6.25, 12.5, 25 *, 50, and 100 µg/mL	Inhibit metastasis through inhibition of tube formation and MMP-9	Park et al. (2016) [48]
	Apple	Pectin	4T1	4T1 (mice)	0.01, 0.1, 0.5, and 1 * % *w*/*v*	Induce apoptosis and inhibit metastasis through up-regulation of p53	Delphi et al. (2016) [49]
	*Capsicum annuum*	CAP	MCF-7 MDA-MB-231 MDA-MB-436	Ehrlich (mice)	0.025, 0.05, 0.1 *, 0.2, 0.4 mg/mL 50, 100 *, and 150 mg/kg	Inhibit proliferation and angiogenesis	Adami et al. (2018) [50]
FUNGUS	*S. Cerevisae*	Β-(1-3)-d-glucan	MCF-7 LCC9	-	1, 10 *, 50 *, 100, 200, 300, and 400 µg/mL	Inhibit proliferation, induce apoptosis and increase genes rassf1, IGFBP3, CTNNB1, and ERβ	Jafaar et al. (2014) [51]
	Botryosphaeria rhodina mamb-05 *Lasiodiplodia theobromae* MMPI	(1-3)(1-6)-β-d-glucan (1-6)-β-d-glucan	MCF-7	-	10, 25, 50, 75, 100 *, 250, 750, 1000, and 1500 µg/mL	Inhibit proliferation, induce apoptosis, necrosis, and oxidative stress mediated by amp-activated protein-kinase and forkhead transcription factor foxo3a	Queiroz et al. (2015) [52]
	*Trichoderma pseudokoningii*	EPS	MCF-7	-	0.10, 0.25 *, 0.50 *, 0.75, and 1 * mg/mL	Induce oxidative stress and apoptosis through an intrinsic mitochondrial pathway	Wang et al. (2015) [53]
	*Aureobasidium pullulan*	Pullulan (PS)	-	4T1 (mice)	2.4 * mg/kg	Immunostimulant of macrophages m1	Xie et al. (2018) [54]
MUSHROOMS	*Pholiota dinghuensis* bi	PDP3	MCF-7	-	13.5, 28.2 *, and 52.1 * µg/mL	Inhibit proliferation and induce apoptosis through p38/MAPK pathway	Gan et al. (2015) [55]
	*Trametes robiniophila murr* *(hauier)*	SP1	MCF-7	-	100 *, 200 *, and 400 * µg/mL	Induce apoptosis through downregulation bax and mtdh protein	Luo et al. (2016) [56]
	*Mortierella hepialid*	MHP-1	MCF-7	MDA-MB-231 (mice)	0.1 *, 1 *, and 10 * µm 20, 40, and 60 * mg/kg	Inhibit metastasis through inhibition of TGF-β signaling	Lin et al. (2016) [57]
	*Lentinus edodes*	Lentinan [β-(1-3)(1-6)-d-glucan]	MCF-7 T47D Mda-mb-231 MAA-MB-468	MCF-7 (mice)	12.5, 25, 50, 100 *, 200 *, and 400 * µg/mL 1 * mg/kg	Inhibited tumor growth through suppressing cell proliferation and enhancing apoptosis via PI3K/AKT/mTOR, NF-κB -, ERK-, ERα-, caspase-, and p53-dependent pathways	Xu, Zou, and Xu (2017) [58]
	*Lentinus edodes*	Lentinan [β-(1-3)(1-6)-d-glucan]	MCF-7	MCF-7 (mice)	15.6, 31.3, 62.5, 125, 250 *, 500 * and 1000 * µg/mL 5 *, 10 *, and 20 * mg/kg	Induce autophagy through LC3 conversion and apoptosis through caspase-7-mediated mitochondrial pathway	Li et al. (2018) [59]

* Represents statistical difference indicated by the author of the study in relation to the control of the experiment. AP-1, activator protein 1; APS, astragalus polysaccharide; Se-PFPs, Se-enriched P. fortuneana; CAP, Capsicum annuum polysaccharide fraction; COX-2, cyclooxygenase 2; DMBA, 7,12-dimethylbenz(a)anthracene; EPS, exopolysaccharide isolated from the fermentation liquor of Trichoderma pseudokoningii fungus; ERK, extracellular signal regulated protein kinase; HIF1A, Polymorphic variation of hypoxia inducible factor-1 A; IGF-1, insulin-like growth factor-1; JNK, c-Jun N-terminal kinase; LBPs, Lycium barbarum polysaccharides; LC3, 1A/1B-light chain 3; LMWF, low molecular weight fucoidan; MAPK, mitogen-activated protein kinase; MMP-9, matrix metalloproteinase-9; NF-κB, factor nuclear kappa B; PD1/PDL1, programmed death-1/programmed death-ligand-1; PDP-3, polysaccharide fraction from Pholiota dinghuensis Bi mycelium; PI3K, phosphatidylin-ositol 3-kinase; Ps, starfish polysaccharide; ROS, reactive oxygen species; TGF-β, transforming growth factor beta; VEGF, vascular endothelial growth factor; WFPs, wolfberry-derived polysaccharides.

## Data Availability

Not applicable.
